# Effects of Focal Low-Energy Extracorporeal Shock Wave Treatment (ESWT) as an Add-On to Botulinum Toxin (BoNT) Injection on Pisa Syndrome in Parkinson’s Disease

**DOI:** 10.5334/tohm.1201

**Published:** 2026-06-04

**Authors:** Paolo Manganotti, Tiziana Maria Isabella Lombardo, Valentina Cenacchi, Mauro Catalan, Giulia Bellavita, Valentina Tommasini, Arianna Sartori

**Affiliations:** 1 Clinical Unit of Neurology, Department of Medicine, Surgery and Health Sciences, University Hospital and Health Services of Trieste, ASUGI, University of Trieste, Strada Di Fiume 447, 34100, Trieste, Italy; 2 Neurology Unit, Hospital Care Department of Medicine, Azienda Sanitaria Universitaria Giuliano Isontina, Trieste, Italy

**Keywords:** ESWT, extracorporeal shock wave therapy, Pisa Syndrome, Parkinson’s disease, botulinum toxin

## Abstract

**Background:**

Pisa syndrome (PS) is a reversible lateral trunk deviation that arises as a complication in a subset of patients with Parkinson’s disease (PD). Effective therapeutic options remain limited, and standardized protocols are lacking. Extracorporeal shock wave therapy (ESWT), an intervention with emerging antidystonic and antispastic properties, has shown promising effects on muscle spasms and dystonia. We aimed at evaluating the feasibility, safety, and preliminary therapeutic effects of ESWT combined with botulinum toxin (BoNT) for reducing paraspinal muscle tone and pain in patients with PS.

**Methods:**

This crossover observational study analyzed data collected in routine clinical practice in patients with PS undergoing focused ESWT applied to ipsilateral paraspinal muscles immediately prior to BoNT injections.

Fifteen patients were enrolled, and eleven completed the full protocol, which included ESWT+BoNT and sham ESWT+BoNT administered according to clinical scheduling, with a washout phase lasting no less than 3 months between treatment conditions. Clinical evaluations were performed at baseline, immediately post-treatment, and at 1 and 3 months post-treatment. Outcome measures included lateral trunk flexion angle, UPDRS part III scores, pain intensity ratings, quality of life assessments, and surface electromyography (sEMG) of axial muscles.

**Results:**

The primary finding was a significant immediate reduction in lateral trunk flexion following active ESWT compared with sham (p = 0.038). However, no sustained effects were observed at 1 or 3 month post-treatment for posture, motor severity, pain, or patient-reported outcomes. sEMG analysis did not demonstrate significant modifications in muscle activation patterns. ESWT was well tolerated, only mild, transient discomfort was reported, and no systemic adverse events occurred.

**Discussion:**

These findings provide evidence that focused ESWT may acutely reduce lateral trunk flexion in PS when used as an adjunct to BoNT. Larger studies are warranted to better define its therapeutic potential.

## Introduction

Parkinson’s disease (PD) is the second most prevalent neurodegenerative disorder worldwide, and its prevalence is projected to rise substantially in the coming decades. In the advanced stages of PD, axial postural deformities, such as Pisa syndrome (PS), camptocormia, antecollis, and scoliosis, represent particularly disabling complications [1]. These conditions are associated with postural instability, an increased risk of falls, pain, and a marked decline in functional autonomy [2]. Pisa syndrome is characterized by a lateral trunk flexion typically exceeding 10° or 15°, depending on the diagnostic criteria adopted, which is reversible in the supine position or with passive mobilization [1,3]. Clinically, PS is evident during sitting, standing, and ambulation, and it generally resolves when the patient lies supine. The onset may be acute, subacute, or chronic; however, in most cases, PS develops insidiously over several months. Patients are frequently unaware of the progressive deviation, leading to underrecognition and underreporting. In the majority of individuals with PD, trunk deviation occurs contralateral to the side of motor symptom onset [4,5]. Clinically, PS is often accompanied by disequilibrium or a subjective sensation of falling toward the leaning side, resulting in a veering gait and significant functional impairment. Pain is commonly reported, typically localized to the lower back and often of moderate to severe intensity [6].

The diagnosis of PS is primarily clinical and relies on the quantification of lateral trunk flexion, which can be assessed using wall-mounted goniometers, inclinometers, or photographic measurements analyzed with dedicated digital software. Beyond clinical assessment, surface electromyography (sEMG) provides valuable insight into muscle activation patterns and may guide therapeutic decision-making. Two principal sEMG patterns have been described: hyperactivation of paraspinal muscles ipsilateral to the bending side, consistent with a dystonic mechanism; and contralateral paraspinal hyperactivation combined with co-activation of non-paraspinal lateral trunk muscles on the leaning side, generally interpreted as a compensatory postural response [7,8].

The management of Pisa syndrome remains challenging due to the absence of standardized treatment guidelines and the limited availability of high quality evidence. Botulinum toxin (BoNT) injections are widely employed, and several case series and small clinical trials have investigated their efficacy in PS. BoNT may attenuate excessive paraspinal muscle hyperactivation; however, treatment response is highly heterogeneous, partly owing to variability in muscle selection, dosing strategies, and injection techniques [9,10]. In a randomized, double blind, placebo controlled crossover trial, Bonanni et al. reported a 50–87% improvement in trunk deviation in six out of nine patients following injections into paraspinal muscles ipsilateral to the bending side [3]. In contrast, Tassorelli et al. adopted a highly individualized approach, relying on EMG-guided identification of hyperactive muscles rather than on the direction of the postural deviation itself, often resulting in bilateral injections; they achieved significant postural improvements and enhanced outcomes when combined with rehabilitation [11]. A recent review proposing a clinical algorithm for the management of axial postural abnormalities in PD further emphasized the importance of individualized, EMG-guided therapeutic strategies, given the marked heterogeneity of muscular activation patterns [12]. Nevertheless, inconsistent outcomes, the lack of standardized protocols, and the potential loss of efficacy over time particularly in chronic forms, may limit the widespread and long-term use of BoNT. Physiotherapy interventions, including postural exercises, stretching, trunk mobility training, proprioceptive stimulation, and functional rehabilitation, have demonstrated variable yet encouraging results. Some studies have reported improvements lasting several months following intensive 4-week rehabilitation programs [13,14,15,16]. However, response rates remain heterogeneous, and the durability of long-term benefits remains uncertain. Overall, both pharmacological and non-pharmacological approaches show variable efficacy, and sustained long-term improvement is not consistently achieved.

Low-energy extracorporeal shock wave therapy (ESWT) is an emerging intervention in neurology, primarily used to reduce spasticity, hypertonia, and pain in conditions such as stroke [17] and dystonia [18]. Preclinical and clinical evidence suggests that ESWT may induce a transient reduction in muscle tone and pain, potentially mediated by decreased acetylcholine release at the neuromuscular junction [19,20]. The aim of the present study was to investigate the effects of low-energy, noninvasive ESWT in patients with advanced PD and Pisa syndrome, with particular focus on its potential to reduce paraspinal muscle tone and pain. This preliminary investigation was designed to assess the feasibility, safety, and initial therapeutic efficacy of ESWT in combination with botulinum toxin as a novel strategy for the management of PS in PD.

## Materials and Methods

### Participants

Patients diagnosed with idiopathic PD presenting with lateral trunk flexion were recruited at the Movement Disorders Clinic of Trieste (Italy) between November 2024 and February 2025. Inclusion criteria were: diagnosis of PD according to the MDS Clinical Diagnostic Criteria [21]; Hoehn & Yahr (H&Y) [22] stage ≥ 2; and dystonic posture consistent with PS based on clinical evaluation and sEMG recordings. Notably, no predefined angular cut-off was required, provided that lateral trunk flexion was clinically relevant (i.e., visible, painful, and/or functionally impairing) and completely reversible in the supine position (clinostatism). Exclusion criteria included concomitant neurological or orthopedic conditions potentially affecting posture (e.g., scoliosis, spondylodiscitis, vertebral fractures, or traumatic spinal injuries), history of major spinal surgery, treatment with medications known to influence posture (e.g., neuroleptics and antiemetics, except for clozapine, quetiapine, and domperidone), changes in dopaminergic therapy within the month preceding baseline evaluation, and BoNT injections in paraspinal muscles within the previous three months.

### Study Design

The study was designed as a crossover observational study involving two groups, based on treatment sequences applied in routine clinical practice. After baseline assessment (T0), participants were allocated to the treatment order according to clinical scheduling.

Group 1 (n = 9) initially received active ESWT treatment + BoNT, followed by sham ESWT treatment + BoNT, after completion of a wash-out phase lasting no less than 3 months. Group 2 (n = 6) received sham ESWT treatment + BoNT first, followed by active ESWT treatment + BoNT after the washout period.

Assessments were performed immediately after treatment (T0*), at 1 month (T1), and at 3 months (T2) in each treatment phase. After the washout period, participants crossed over to the alternative treatment condition and repeated the same assessment schedule.

**Figure 1:**
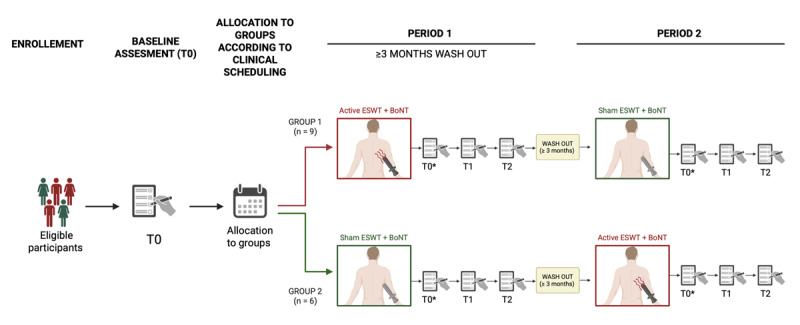
Study protocol and timeline. After enrollment, all participants underwent baseline assessment (T0) and were then allocated to one of two treatment sequences according to clinical scheduling: Group 1 (active ESWT + BoNT followed by sham ESWT + BoNT) or Group 2 (sham ESWT + BoNT followed by active ESWT + BoNT). Each treatment period included an immediate post-treatment assessment (T0*), followed by follow-up evaluations at 1 month (T1) and 3 months (T2). A washout period of at least 3 months separated the two treatment periods, after which participants crossed over to the alternate intervention. This design allowed within-subject comparisons between active and sham conditions. ESWT = extracorporeal shock wave therapy. BoNT= Botulinum toxin.

All participants underwent both treatment conditions in randomized order, allowing for within-subject comparisons (Figure 1).

### ESWT Protocol

**Figure 2:**
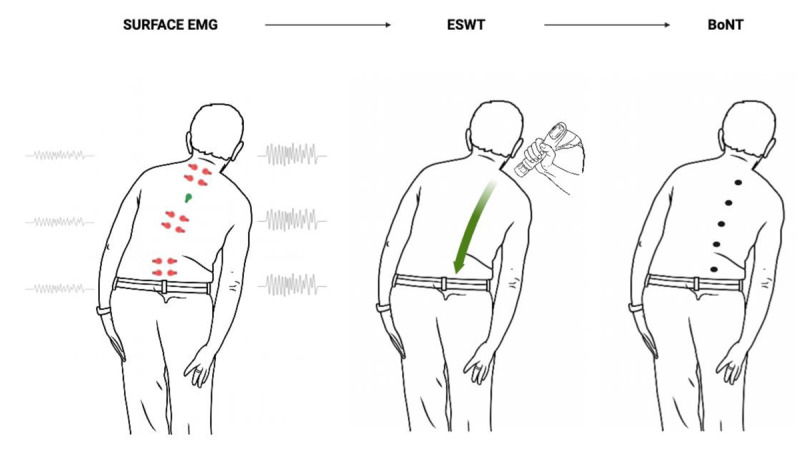
Schematic representation of focused ESWT applied prior to BoNT injection, illustrating active stimulation of the paraspinal muscles in the treatment condition (see Figure 3 for a real image example), and sham stimulation with minimal energy and no direct tissue contact in the placebo condition.

**Figure 3:**
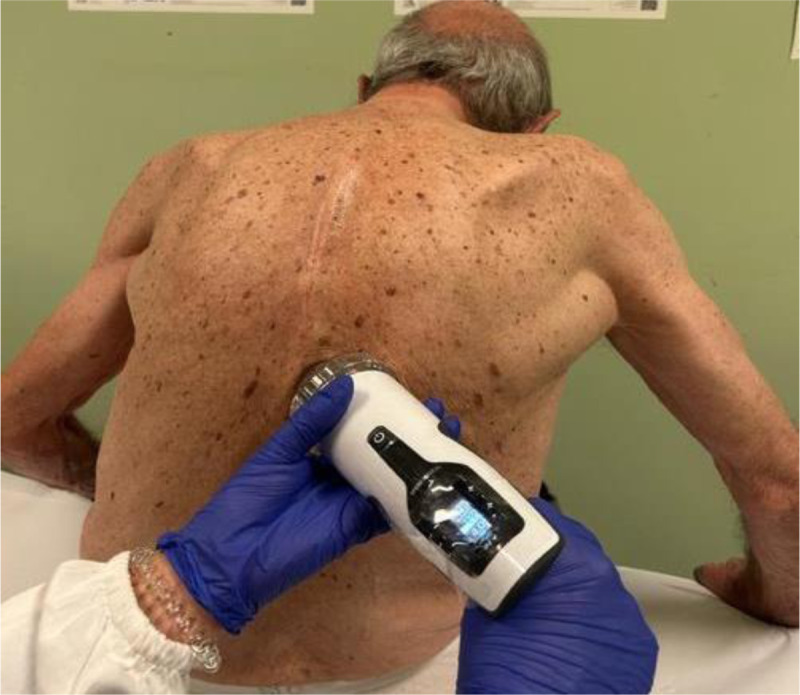
ESWT application to paraspinal muscles.

Patients assigned to the active treatment condition received a single session of focused ESWT administered 10 minutes prior to BoNT injection, targeting the paraspinal muscles ipsilateral to trunk flexion. Each ESWT session consisted of 3000 impulses delivered at an energy flux density of 0.30 mJ/mm² and a frequency of 4 Hz. In the placebo condition, sham ESWT stimulation was administered prior to BoNT injection, delivering 3000 impulses at an energy flux density of 0.01 mJ/mm². An additional air cushion was interposed between the shockwave generator and the applicator to prevent effective transmission of shock waves to the paraspinal muscles, thereby ensuring the absence of therapeutic stimulation. From the patient’s perspective, however, the sham procedure was indistinguishable from real stimulation, as full contact between the shockwave applicator and the patient’s back was maintained (Figures 2 and 3).

### BoNT Injection Protocol

All patients first received ESWT (either active or sham), applied to the paraspinal muscles ipsilateral to the bending side, followed by BoNT injections administered to the same regions from the dorsal to the lumbar levels. The treatment sequence was specifically designed to minimize uncertainty regarding potential interactions between ESWT and BoNT. Although ESWT is known to modulate tissue perfusion and cellular activity, its possible effects on the distribution or stability of pharmacological agents remain unclear. Therefore, ESWT was delivered prior to BoNT injections to avoid potential alterations in toxin efficacy.

With regard to BoNT injections, a total of 100 International Units (IU) of Onabotulinumtoxin A, diluted to 50 IU/mL, were injected into five sites along the paraspinal muscle group ipsilateral to the side of trunk deviation.

### Clinical and Instrumental Assessments

Patients were evaluated at baseline (T0), immediately after treatment (T0*), and at one month (T1) and three months (T2) following BoNT injection and the ESWT session (either active or sham). Lateral trunk flexion angle was measured on planar-view photographs using the online software calculator NeuroPostureApp (https://www.neuroimaging.uni-kiel.de/NeuroPostureApp/). Clinical assessments included the Unified Parkinson’s Disease Rating Scale (UPDRS) [23], the Parkinson’s Disease Questionnaire-8 (PDQ-8) [24], a 1-to-10 Numeric Rating Scale (NRS) for pain, and the Patient Global Impression of Change (PGI-I 5, PGI-I) scale [25], alongside global clinical evaluation, including presence of Freezing of Gait (FOG severity score). An eight-channel surface electromyograph (EMG) recorded bilateral activity of the paraspinal muscles at cervical (C6–C7), thoracic (T8–T10), and lumbar (L2–L4) levels, as well as the oblique abdominal muscles. Recordings were performed while simultaneously video-recording patients in supine, sitting, and standing positions, both at rest and during active trunk flexion, according to previously published protocols [7]. sEMG traces were analyzed both qualitatively and quantitatively. For quantitative analysis, mean amplitude and frequency values were compared between baseline and follow-up evaluations at T1 and T2.

### Statistical Analysis

The Shapiro-Wilk test was used to assess the normality of continuous data distribution. Continuous variables were expressed as mean ± standard deviation (SD) or median (range), as appropriate. Between-group comparisons of continuous variables were performed using the Student’s *t*-test or the Mann-Whitney *U* test, depending on data distribution. Categorical variables were analyzed using Pearson’s chi-square test or Fisher’s exact test, as appropriate. To evaluate the effects of treatment on clinical scores over time, a general linear model (GLM) for repeated measures was applied, with Time (baseline and post-treatment) as the within-subject factor and Treatment (sham vs. ESWT) as the between-subjects factor. Treatment order was included as a covariate. No additional covariates were entered into the model, as the groups did not differ significantly in baseline characteristics. Post hoc analyses were performed using Bonferroni correction for multiple comparisons. All statistical analyses were conducted using IBM Corp. SPSS Statistics for Mac, Version 31.0.

## Results

### Baseline Clinical Characteristics

Patients’ clinical characteristics are reported in Tables 1 and 2. No statistically significant differences were observed between the two groups according to treatment order. Five patients in Group 1 and six patients in Group 2 completed the study. Baseline characteristics of the patients who completed the protocol were comparable between groups, with no statistically significant differences detected.

**Table 1:** Patients’ characteristics.


	GROUP 1 (ESWT → sham) n = 9	GROUP 2 (sham → ESWT) n = 6	p

Sex, F	3 (33.3%)	2 (33.3%)	1.000^a^

Age, y	78.0 ± 4.3	74.3 ± 2.7	0.058^b^

Height, m	1.70 ± 0.07	1.71 ± 0.06	0.783^b^

Weight, kg	70 (56–97)	74.2 (60–102)	0.906^c^

BMI	24.3 ± 3.1	25.4 ± 4.1	0.585^b^

PD age onset, y	68.8 ± 7.5	67.2 ± 3.0	0.575^b^

Disease duration, y	9.2 ± 4.6	6.5 ± 2.8	0.218^b^

PD latency, y	5.4 ± 3.6	3.8 ± 3.1	0.383^b^

Treatment latency, y	1 (0.8–2)	1.25 (0.5–3)	0.671^c^

PS duration, y	3.67 ± 1.66	3.17 ± 2.56	0.652^b^

Phenotype			0.460^d^

Tremor dominant	1 (6.7%)	1 (16.7%)	

Rigid-akinetic	2 (22.2%)	0	

Mixed	6 (66.7%)	5 (83.3%)	

Side of symptoms			0.329^a^

Right	7 (77.8%)	3 (50%)	

Left	2 (22.2%)	3 (50%)	

Falls	1 (11.1%)	0 (0%)	1.000^a^

Comorbidity (n = 9, n = 5)	9 (100%)	5 (100%)	na

Rehabilitation within last 3 months	2 (22.2%)	2 (33.3%)	1.000^a^

PS direction at baseline			0.608^a^

Right	5 (55.6%)	2 (33.3%)	

Left	4 (44.4%)	4 (66.7%)	

Metronome phenomenon	1 (11.1%)	0 (0%)	1.000^a^

PS pattern at onset			1.000^a^

Chronic	8 (88.9%)	6 (100%)	

Subacute	1 (11.1%)	0 (0%)	

PS side at onset			0.580^a^

Ipsilateral	4 (44.4%)	1 (16.7%)	

Contralateral	5 (55.5%)	5 (83.3%)	

Awareness	7 (77.8%)	5 (83.3%)	1.000^a^

Head compensation	2 (22.2%)	4 (66.7%)	0.136^a^

Sensory trick	0 (0%)	0 (0%)	na


^a^Fisher’s Exact Test; ^b^Independent samples T-test; ^c^Mann Whitney U; ^d^Chi square Test; na = not applicable.

**Table 2:** Clinical scores at baseline.


	GROUP 1 (ESWT → sham) n = 9	GROUP 2 (sham → ESWT) n = 6	p

Pisa syndrome degrees	8.33 ± 4.31	8.85 ± 4.65	0.829^b^

LEDD	666.7 ± 222.1	568.7 ± 196.40	0.398^b^

FOG, n (%)	5 (55.6%)	1 (16.7%)	0.287^a^

FOG severity	5.44 ± 6.37	1 ± 2.45	0.085^b^

PDQ8	4.22 ± 2.11	5.17 ± 5.12	0.625^b^

H&Y	2 (2–3)	2 (2–3)	0.776^c^

Pain, n (%)	4 (44.4%)	3 (50%)	1.000^a^

NRS other than back	0 (0–7)	0 (0–0)	0.776^c^

NRS back	0 (0–7)	1 (0–4)	1.000^c^

UPDRS-III	31.89 ± 8.21	36.67 ± 17.72	0.558^b^


^a^Fisher’s Exact Test, ^b^Independent samples T-test, ^c^Mann Whitney U.

*Treatment 1* indicates patients who received sham ESWT, whereas *Treatment 2* included patients who received active ESWT.

Baseline (T0) scores did not show statistically significant differences between Treatment 1 (sham) and Treatment 2 (ESWT). Similarly, along the study, we observed no changes in PD severity (in terms of UPDRSIII, H&Y scale), or L-Dopa treatment (LEDD) as expected (data not shown).

**Figure 4:**
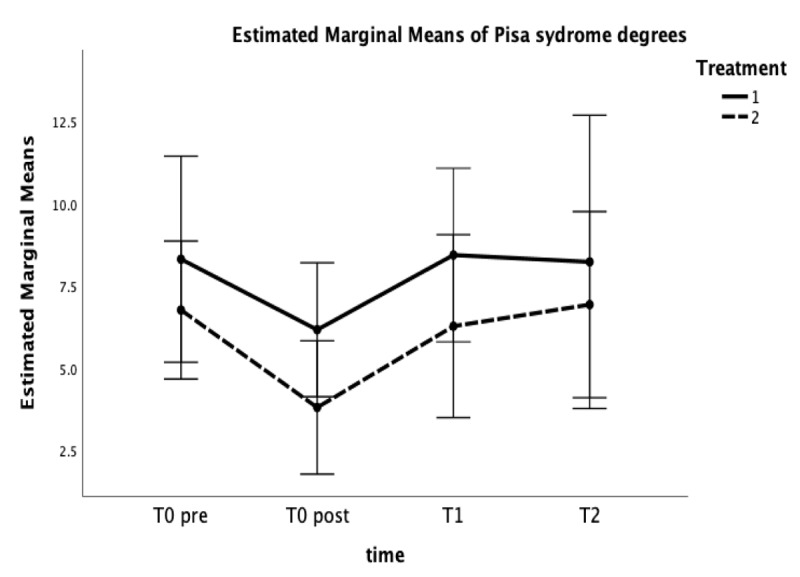
Angular measurements across the different study time points. Treatment 1 = sham; Treatment 2 = ESWT; Error Bars: 95% CI.

**Figure 5:**
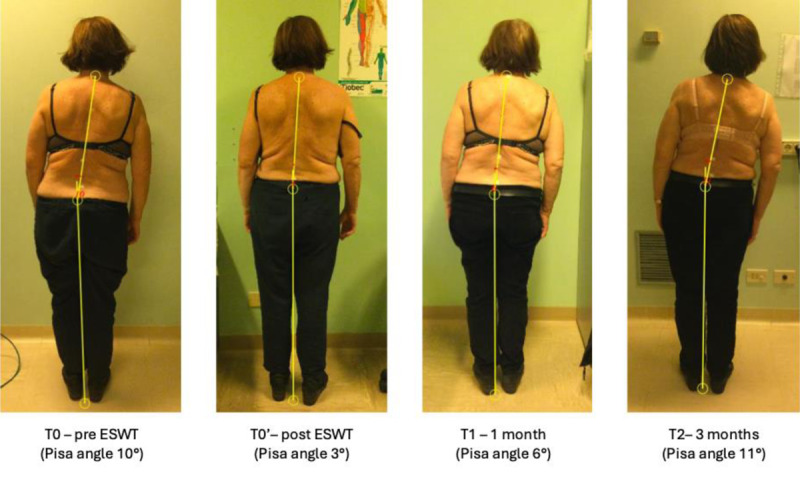
Representative image of a study patient illustrating the angular variation at T0, T0’, T1, and T2.

For Pisa syndrome degrees, repeated measures GLM revealed a significant main effect of Treatment, *F*(3, 27) = 3.54, *p* = 0.037, partial *η^2^* = 0.399, but no effect for Time main factor, for the Treatment × Order, Time × Order, Treatment × Time, and Time × Treatment × Order interactions. Considering pairwise comparisons, the only statistically significant differences were observed in Treatment 2 (ESWT) T0-pre vs T0-post, with a mean difference of 2.96 (95% CI 0.32 to 5.51), p = 0.027). Moreover, at T0-post Treatment 2 showed statistically significant lower degrees of Pisa syndrome compared to Treatment 1, with a mean difference of 2.36 (95% CI –4.57 to –0.16), p = 0.038 (Figure 4). A representative example of angular variation across time points (T0, T0*, T1, and T2) is shown in Figure 5.

**Figure 6:**
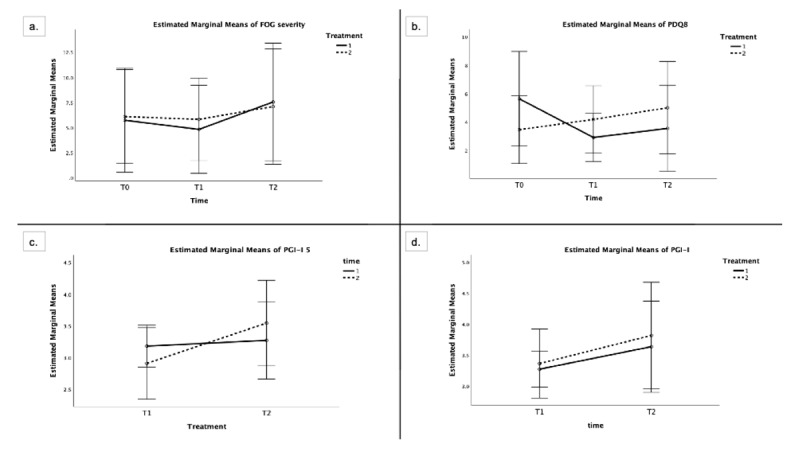
FOG severity score, PDQ-8, PGI-I (5-point), PGI-I total score, and NRS pain scores across time. Repeated-measures GLM showed no significant effects of Time, Treatment, or Time × Treatment interaction. No significant post-treatment vs baseline differences were observed in either group **(panels a–d)**. Treatment 1 = sham; Treatment 2 = ESWT; Error Bars: 95% CI.

**Figure 7:**
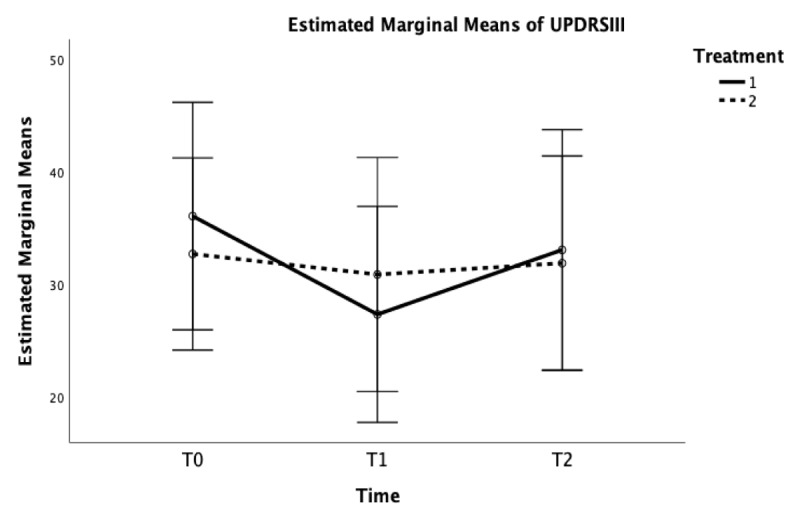
UPDRS-III scores across time and treatment conditions. Treatment 1 = sham; Treatment 2 = ESWT; Error Bars: 95% CI.

For the FOG severity score, PDQ8, PGI-I 5, PGI-I scores, and NRS for pain scores, GLM for repeated measures did not reveal a significant main effect of Time, Treatment or for the Time×Treatment interaction. No significant differences were observed between post-treatment and baseline values for both the treatment groups (Figure 6, panel a-d). For UPDRS-III, repeated measures GLM revealed a significant main effect of Time, *F*(1, 18) = 4.46, *p* = 0.027, partial *η^2^* = 0.331, but no effect for Treatment main factor, for the Treatment×Order, Time×Order, Treatment×Time, and Time×Treatment×Order interactions. Considering pairwise comparisons, the only statistically significant differences were observed in Treatment 2 (sham) T0 pre vs T0 post, with a mean difference of 8.73 (95% CI 0.47 to 16.98), p = 0.038 (Figure 7).

### sEMG Analysis

Two patients (one from each treatment-order group) were excluded from the sEMG analysis due to tremor-related artifacts. Minimum, maximum, mean, and delta sEMG activity were analyzed at both dorsal and lumbar levels, on the bending side and contralateral side. The general linear model (GLM) for repeated measures did not reveal a significant main effect of Time or Treatment, nor a significant Time×Treatment interaction for any of the analyzed sEMG parameters. Furthermore, no significant differences were observed between post-treatment and baseline values in either treatment condition. Qualitative assessment of sEMG recordings showed that most patients predominantly exhibited pattern II, characterized by contralateral compensatory activity.

## Discussion

The main finding of this study was a significant and immediate reduction in lateral trunk flexion following a single session of active ESWT compared with sham stimulation. In the active treatment condition, the Pisa angle significantly decreased from T0-pre to T0-post, with a mean reduction of approximately 3°, and the post-treatment angle was significantly lower after ESWT than after sham. However, no sustained differences were observed at the 1- and 3-month follow-up assessments with respect to trunk angle, UPDRS-III scores, pain intensity, or quality of life. In addition, surface EMG analysis did not demonstrate significant changes in paraspinal muscle activation patterns at the group level. Overall, this single session protocol proved to be feasible and well tolerated, with only minimal and transient discomfort reported during stimulation by a small number of patients and no systemic adverse effects observed.

The rapid yet transient effect of ESWT on paraspinal dystonia is consistent with previous evidence showing that ESWT can temporarily reduce muscle hypertonia and modulate soft-tissue mechanical properties in hypertonic conditions of various etiologies, including stroke, cerebral palsy, multiple sclerosis, other upper motor neuron syndromes, and dystonia [17,18,26,27]. Several randomized controlled trials and recent systematic reviews have documented significant reductions in spasticity scores (e.g., Modified Ashworth Scale, Tardieu Scale) and improvements in range of motion following ESWT, with effects lasting from several weeks to a few months depending on treatment parameters and muscle characteristics [27,28]. Furthermore, studies in focal dystonia suggest that ESWT may reduce dystonic muscle overactivity without inducing muscle weakness, likely through effects on muscle structure and metabolic processes rather than direct modulation of neural transmission [18]. Our findings extend these observations to axial dystonic and postural deformities in PD, demonstrating that ESWT can acutely reduce lateral trunk flexion in patients with PS. Nevertheless, in the present protocol the effect was short-lived and did not translate into sustained improvements in posture or global disability. Several factors may account for this limited durability, including the administration of a single ESWT session, the exclusive targeting of paraspinal muscles, and the relatively mild baseline trunk deviation in our cohort (mean 8–9°), which included both early and chronic forms of Pisa syndrome. It is plausible that repeated ESWT sessions, alternative energy settings, or broader muscle targeting could induce more durable structural and functional adaptations. sEMG analysis did not reveal any significant changes in paraspinal muscle activity; however, given the small sample size, the technical challenges inherent in recording from deep paraspinal musculature, and the modest magnitude of trunk angle change, subtle neurophysiological alterations cannot be excluded and may not have been captured by our assessment methods. Moreover, the acute mechanical and neuromodulatory effects of ESWT may precede, or occur independently of, detectable changes in resting EMG parameters, particularly following a single treatment session.

A key methodological decision in our protocol was to apply one session of ESWT prior to BoNT administration rather than immediately afterward. The combined use of ESWT and BoNT has previously been explored in a clinical trial in which ESWT delivered after BoNT was shown to be safe and potentially synergistic in the treatment of post-stroke limb spasticity [29]. However, ESWT is known to modulate tissue perfusion, cell membrane permeability, inflammatory mediators, and possibly neuromuscular junction stability [30,31]. It is therefore conceivable, although not yet demonstrated, that applying ESWT directly over and after a recent BoNT injection site could alter toxin distribution, potentially affecting its molecular stability. Such mechanisms could theoretically reduce focal efficacy or increase the risk of adverse effects. Given that the interaction between shock waves and BoNT at the injection site remains insufficiently understood, particularly in axial musculature, we adopted a conservative approach by delivering ESWT prior to BoNT administration. This strategy was intended not only to avoid potential interference with toxin action but also to “precondition” the muscle tissue. In this context, ESWT may reduce local fibrotic changes within the muscle, thereby potentially facilitating subsequent BoNT diffusion and effectiveness. We elected to treat exclusively the paraspinal muscles ipsilateral to the direction of trunk bending, irrespective of individual EMG patterns, based on pathophysiological considerations and methodological consistency. Previous EMG and BoNT studies suggest that the primary dystonic drive in Pisa syndrome most frequently originates from ipsilateral paraspinal muscles, whereas contralateral hyperactivity is more likely compensatory [7]. Targeting the ipsilateral side allowed us to focus on the muscles most plausibly responsible for the deformity, avoid unnecessary weakening of compensatory contralateral muscles, and maintain a standardized and reproducible treatment protocol. Although EMG analysis did not reveal significant group-level changes, the acute clinical improvement observed following ipsilateral ESWT supports the hypothesis that these muscles play a primary role in lateral trunk flexion, in line with current pathophysiological models [12]. ESWT was generally safe and well tolerated. A small number of patients reported mild, transient local discomfort during stimulation, consistent with previous studies investigating ESWT for spasticity and tendinopathies [32,33].

Several limitations should be acknowledged. The sample size was small, with only 11 patients completing the full crossover protocol, thereby limiting statistical power and generalizability. The use of a single ESWT session may underestimate the therapeutic potential of this intervention. In addition, the crossover design combined with a relatively short washout period raises the possibility of residual carry-over effects. Only patients with mild to moderate deformity were included, limiting the applicability of our findings to more severe cases. Furthermore, ESWT and BoNT were not systematically combined with a standardized rehabilitation program, potentially restricting consolidation of the observed acute benefits. Accordingly, our findings should be interpreted as preliminary and exploratory rather than definitive evidence of efficacy. Future studies should include larger randomized cohorts and evaluate repeated ESWT sessions with systematic variation of energy levels, number of impulses, and treatment intervals. Furthermore, it may be worthwhile to explore alternative timing protocols: for example, administering ESWT approximately one month after BoNT (when the toxin’s effect is at its peak) to determine whether this approach could further potentiate the combined therapeutic impact. Multimodal approaches integrating ESWT, BoNT, and structured rehabilitation programs should be explored to enhance and stabilize axial realignment. Extending ESWT application to other axial muscle groups, including cervical musculature, may clarify its potential utility in other postural deformities such as antecollis. Finally, patient stratification according to clinical phenotype, such as early versus chronic Pisa syndrome, mobile versus fixed deformities, and distinct EMG patterns (e.g., Pattern I vs. Pattern II), may help identify subgroups most likely to benefit from ESWT.

## Conclusions

This crossover study provides the first evidence that focused ESWT targeting ipsilateral paraspinal muscles in patients with Parkinson’s disease and Pisa syndrome is feasible, safe, and capable of inducing an immediate, albeit transient, reduction in lateral trunk flexion when used as an adjunct to botulinum toxin treatment. The absence of clear medium-term benefits, together with the lack of significant EMG changes at the group level, suggests that a single ESWT session is insufficient to achieve sustained axial realignment in chronic Pisa syndrome. Nevertheless, the novelty of the observed acute effects supports the concept that ESWT can modulate chronically hyperactive and structurally altered paraspinal muscles, consistent with its established anti-spastic and antidystonic properties in other neurological conditions. Collectively, these findings indicate that ESWT represents a promising, non-invasive adjunctive intervention within a multidisciplinary management strategy for Pisa syndrome in Parkinson’s disease, warranting further investigation through optimized treatment protocols, larger patient cohorts, and integrated rehabilitative approaches.

## Generative AI statement

The author(s) declared that Generative AI was not used in the creation of this manuscript.

## Data Availability

The data supporting this study’s findings are available from the corresponding author upon reasonable request.
